# HLA‐E: Immune Receptor Functional Mechanisms Revealed by Structural Studies

**DOI:** 10.1111/imr.13434

**Published:** 2025-01-03

**Authors:** Geraldine M. Gillespie, Max N. Quastel, Andrew J. McMichael

**Affiliations:** ^1^ Nuffield Department of Medicine, Center for Immuno‐Oncology University of Oxford Oxford UK

**Keywords:** CD8 T cells, HLA‐E, MHC class I, MHC‐E, NKG2A, T‐cell receptors

## Abstract

HLA‐E is a nonclassical, nonpolymorphic, class Ib HLA molecule. Its primary function is to present a conserved nonamer peptide, termed VL9, derived from the signal sequence of classical MHC molecules to the NKG2x‐CD94 receptors on NK cells and a subset of T lymphocytes. These receptors regulate the function of NK cells, and the importance of this role, which is conserved across mammalian species, probably accounts for the lack of genetic polymorphism. A second minor function is to present other, weaker binding, pathogen‐derived peptides to T lymphocytes. Most of these peptides bind suboptimally to HLA‐E, but this binding appears to be enabled by the relative stability of peptide‐free, but receptive, HLA‐E‐β2m complexes. This, in turn, may favor nonclassical antigen processing that may be associated with bacteria infected cells. This review explores how the structure of HLA‐E, bound to different peptides and then to NKG2‐CD94 or T‐cell receptors, relates to HLA‐E cell biology and immunology. A detailed understanding of this molecule could open up opportunities for development of universal T‐cell and NK‐cell‐based immunotherapies.

## 1 Introduction

Alongside the intensely studied highly polymorphic peptide presenting classical major histocompatibility complex (MHC) molecules, there are discrete nonclassical class Ib MHC proteins that are genetically nonpolymorphic. In humans, these are HLA‐E, HLA‐F, HLA‐G, HFE, and MR1. There are also MHC‐like proteins that include CD1a, CD1b, CD1c, and CD1d. Several of these nonclassical MHC molecules appear to be important in both innate immunity and anti‐bacterial immunity. They bind a broad range of antigens that include peptides (HLA‐E, HLA‐G), B vitamin metabolites (MR1) [1], and lipids (CD1) [2]. One nonclassical HLA class Ib molecule that has stimulated much recent interest is HLA‐E and its equivalents in mice and rhesus monkeys, H‐2 Qa1b and Mamu‐E, respectively. These molecules are characterized by low expression on cell surfaces and very limited genetic polymorphism [3]. HLA‐E, which is the subject of this review, contributes to both innate and adaptive immunity. The former is probably most crucial, and likely accounts for its nonpolymorphism, the latter may be rarely used in combatting virus infections, but might be important in some bacterial infections, and now offers exciting opportunities for immunotherapy.

## 2 Cell Biology of HLA‐E

HLA‐E is expressed at low levels on the surface of cells, but it is at least four times more abundant in the endoplasmic reticulum (ER) [4]. In humans there are two major alleles, E*01:01 and E*01:03, which are both present at a similar frequency [5]. The dimorphism is a single‐amino acid change at position 107 outside the peptide‐binding groove (PBG), Arg for E*01:01 and Gly for E*01:03. This dimorphism affects the level of HLA‐E surface expression: E*01:03 is expressed at higher levels than E*01:01 [5]. There may also be an effect on peptide content [6, 7, 8, 9] or stability [5], but there is no difference in the crystal structure of the peptide‐binding groove [5]. Although functional differences are uncertain, E*01:03 has been shown to protect against severe forms of symptomatic infectious mononucleosis caused by Epstein–Barr virus (EBV) [10].

The discovery of the dominant peptide ligand for MHC‐E was made by Aldrich et al. [11]. Studying alloreactive T‐cell clones in mice, they found an H‐2 Qa‐1b‐restricted T‐cell response to a peptide (AMAPRTLLL) that was derived from the signal sequence of H‐2K and D molecules. It was then found that HLA‐E binds the equivalent peptide VMAPRTLVL derived from HLA‐A*02 or similar peptides derived from other HLA‐A, B, C, or G molecules, which show minor conservative variations at residues 6, 7, or 8 [12]. About half of HLA‐B allotypes have a Thr instead of Met at position 2 in this signal peptide which abrogates binding to HLA‐E [13]. In order to determine the nature of a possible receptor for HLA‐E, Braud et al. [13] produced HLA‐E tetramers with the VL9 peptide, and showed that it bound to the NKG2A/C‐CD94 proteins on natural killer (NK) cells, and a subset of CD8 T cells, also shown by Lee et al. [14]. Furthermore, it was shown that the signal sequence of classical class I MHC proteins was processed by a signal peptide peptidase, and then entered the transporter associated with antigen processing (TAP)‐dependent classical peptide processing pathway [15] to bind to HLA‐E. In normal cells, more than 80% of HLA‐E bound peptides comprise this peptide [16] and trafficking of HLA‐E from the ER to the cell surface depends on the supply of this peptide [4]. That supply is normally limiting, so that the bulk of HLA‐E is actually located within the ER rather than the cell surface. Interaction between HLA‐E‐VL9 and the NKG2A inhibitory receptor has higher affinity than binding to the activating receptor, NKG2C [17], and NK‐cell killing of cells that express HLA molecules is therefore inhibited. Viruses that downregulate HLA class I including human cytomegalovirus (HCMV) [18], human immunodeficiency virus 1 (HIV‐1) [18], and severe acute respiratory syndrome coronavirus‐2 (SARS‐CoV‐2) [19] spare HLA‐E so that this NK inhibitory pathway is maintained, to the advantage of the virus. Cancers that frequently downregulate classical HLA molecules to evade lytic T‐cell responses often upregulate HLA‐E, likely selected by their resistance to NK‐cell attack. Under normal circumstances, however, the low expression of HLA‐E and the binding dominance of the MHC class I signal peptide means that HLA‐E‐restricted T‐cell responses, directed at other HLA‐E‐binding peptides, are rarely primed in virus infections.

At the cell surface, HLA‐E has a short half‐life of about 12 min compared to several hours for classical HLA class I molecules [20, 21]. HLA‐E is rapidly internalized and traffics to endosomes, where it has been hypothesized that it acquires lysosome‐digested, non‐VL9 peptides, and recycles to the cell surface [4, 18]. This has some similarities to the MHC class II antigen‐processing pathway where newly synthesized class II molecules in the ER bind to a chaperone, invariant chain (CD74), that occupies the peptide binding groove and thereby enables its cytoplasmic domain to direct the class II protein to the cell surface, where it then internalizes to endosomes [22]. There the invariant chain is digested away and other peptides, generated in lysosomes, bind to the class II molecule, enabling it to recycle to the cell surface to stimulate CD4 T cells.

Mamu‐E traffics in a similar way and priming of Mamu‐E‐restricted SIV‐specific T‐cell responses by a cytomegalovirus (RhCMV68‐1)‐vectored vaccine was found to be dependent on prior trafficking of Mamu‐E to the cell surface with the signal peptide and then endosomal peptide exchange [18]. This MHC‐E‐restricted T‐cell priming process was reliant on RhCMV69‐1 infection of myeloid cells [23], which may be uniquely able to prime T cells in this way. In these RhCMV‐vectored vaccine experiments in monkeys, the CMV‐mediated downregulation of competing classical MHC molecules and the restricted tropism of that particular strain of CMV are likely to have created the special conditions necessary to generate such T‐cell responses. These T cells were capable of clearing SIV in the early stages after experimental challenge infection [24, 25]. It is unclear exactly how this unique clearance of an infecting lentivirus, SIV, was achieved, nor why this only happened in 50%–60% of monkeys in each experiment [24, 25]. The cytokine environment, particularly overexpression of IL‐15 likely contributes to the outcome [26], but protection is undoubtly mediated by MHC‐E‐restricted SIV‐specific CD8+ T lymphocytes.

A second situation where MHC‐E‐restricted T cells are found is in human mycobacterial infection [27]. Nearly all humans who have been infected with one or more environmental mycobacteria, or vaccinated with BCG, show detectable HLA‐E‐restricted mycobacterial peptide‐specific T‐cell responses. This has been estimated to represent the majority of the CD8 T‐cell response to this bacterial infection [28]. HLA‐E‐restricted T cells have also been described in infections associated with *Salmonella* [29], hepatitis C virus (HCV) [30], hepatitis B virus (HBV) [31], HCMV [32], EBVs [10], HIV‐1 [33, 34], and SARS‐CoV‐2 [35].

Mycobacterial and *Salmonella* infections are intracellular and are likely to involve macrophages. For mycobacteria, the bacteria reside in phagolysosomes and HLA‐E‐peptide presentation has been shown to involve recycling HLA‐E molecules, rather than the classical TAP‐dependent ER‐dependent antigen‐processing pathway [36]. The abundance of macrophages in the liver and airways might help to explain why HLA‐E‐restricted T cells have been found in HBV and SARS‐CoV‐2 infections.

HLA‐E and its nonhuman primate and murine equivalents therefore play a critical role in innate immunity and an increasingly recognized, though still minor, role in adaptive immunity to viral and bacterial pathogens. As will be discussed at the end of this review, they also show considerable translational potential. Despite extensive characterization of their biological functions, there have been many puzzling findings, particularly contrasting to classical HLA molecules. However, many of these findings have become more explicable following studies of HLA‐E biochemistry and molecular structure, the main topic of this review.

## 3 Identification of HLA‐E Binding Peptides

The classical route to finding MHC class I‐binding epitope peptides has been to dissect a T‐cell response, first identifying the source protein, then searching or predicting peptides, and then demonstrating T‐cell recognition [37], often using MHC–peptide multimers. Given the apparent rarity of MHC‐E‐restricted T‐cell responses in most situations, we have designed three independent peptide‐binding assays to identify HLA‐E binding peptides. The first is a cell‐based method, known as the single‐chain trimer (SCT) method where the test peptide is fused to β2m and the HLA‐E heavy chain (hc) as a DNA construct which is then transiently transfected into HEK293T cells [25, 38]. The level of cell‐surface expression serves as a proxy for binding strength and is measured using the HLA‐E‐specific 3D12 antibody that recognizes a discontinuous region within the α3 domain [25, 39]. The second approach involves a peptide‐exchange sandwich‐ELISA method using HLA‐E and β2m chemically folded with a UV‐sensitive VL9 peptide (VL9^UV^) with the solvent‐exposed Arg at position 5 replaced by a 3‐amino‐3‐(2‐nitrophenyl)‐propionic acid residue [40]. When subsequently incubated with a nonbinding peptide, the HLA‐E‐β2m–VL9 UV complexes disintegrate either upon 1 h photoillumination or without illumination if incubated overnight at 4°C [40]. However, the HLA‐E complex can be rescued in the presence of a binding peptide, and the strength of a sandwich ELISA signal, that relies on heavy chain capture (via 3D12) and β2m detection, reflects the degree of HLA‐E complex recovery and thus is a surrogate for peptide‐binding strength. The third high‐throughput method is based on the assessment of thermal stability (thermal melt temperature, Tm) of peptide–HLA‐E‐β2m complexes using nano‐differential scanning fluorimetry (nDFS) following incubation of folded (peptide‐free) HLA‐E‐β2m with a molar excess of test peptides [41]. These three independent assays provide a very reliable and quantitative measure of peptide binding, and successfully establish peptide‐binding hierarchies that show strong correlations when compared to each other [41]. The VL9 signal peptide is typically the strongest signal in these three assays. Certain points should be considered when relating the peptide‐binding strength obtained using these assays to a strong binder such as VL9. In the SCT assays, the test peptide is tethered to β2m ‐ this allows peptide rebinding and could, for some peptides, give an exaggerated measure of binding strength compared to nonlinked peptide. The peptide exchange sandwich ELISA method on the other hand gauges peptide binding following an incubation period where test peptide is removed. However, despite internal normalization to express binding as a percentage of VL9 binding, there is a degree of assay‐to‐assay variation that is most evident for weaker binding peptides. The nDSF‐based method is an exquisitely sensitive and highly reproducible assay [40]. However, caution should be applied when testing peptides that contain Cys residues since these can oxidize upon heat treatment resulting in an underestimate of Tm values if the residues are directly involved in peptide binding. When peptide‐free HLA‐E‐β2m complexes are incubated with molar excess of medium/low‐strength binding peptides, the measured Tm values show nonlinear differences to HLA‐E‐β2m–peptide complexes purified away from excess peptides prior to evaluation (referred to here as “pre‐refolded” complexes). However, the granularity of the method improves substantially when lower concentrations of peptide, 10 molar excess or less, are tested. These differences reflect the very short half‐lives of many peptides, including viral epitopes, that bind HLA‐E (half‐life of minutes), compared to VL9 sequences (half‐life > 3 h) [20]. The establishment of affinity‐based measurements using either biochemical (e.g., nDSF) [42], native mass spectrometry (nMS) [43], fluorescent polarization (FP) [44], or cellular‐based approaches should provide opportunity to measure the fine details of peptide binding to HLA‐E and to assess how this equates to the various peptide‐binding assays.

Despite not providing more nuanced measures such as binding affinities, the methods described above provide very reliable confirmation of peptide binding and offer a reliable gauge of relative binding strengths. Information from these assays can also be successfully combined with a simple workflow to predict epitope discovery—the peptides identified can be used to prime T cells in vitro or boost existing weak responses. This strategy has successfully led to the identification of bona fide viral epitopes restricted by HLA‐E on virally infected cells [34, 35]. Alongside the publicly available peptide prediction tools (e.g., NetMHC), we also include a simple HLA‐E‐binding motif generated in‐house using binding data acquired through the HLA‐E‐based UV peptide exchange‐sandwich ELISA for over 100 peptides [40]. The in‐house motif integrates criteria based on wet‐laboratory data and extends beyond information available in neural network‐based algorithms. Apart from preferences for Met or Leu at position 2 and Leu at the C terminus of the peptide, this motif extends to the inclusion of specific amino acids that compensate for a lack of preferred anchor residues, most importantly Pro residues at positions 3–7 of the peptide, and tolerability for specific residues, for example, position 3 of the peptide, beyond that predicted from the original x‐ray structures of HLA‐E in complex with VL9 peptides [40, 41, 45]. We have successfully used a number of HIV‐ and SARS‐CoV2‐derived peptides, proven to bind to HLA‐E, to prime T cells from healthy, naïve donors in vitro [34, 35]. We have also identified CD8+ T‐cell responses against HLA‐E‐restricted SARS‐CoV2 Spike and nonstructural (NS) epitopes in convalescent COVID19 patients [34, 35], thus validating that this approach can reliably identify epitopes that are naturally presented by HLA‐E in vivo.

## 4 Structural Analysis of HLA‐E in Complex With Peptide

**FIGURE 1:**
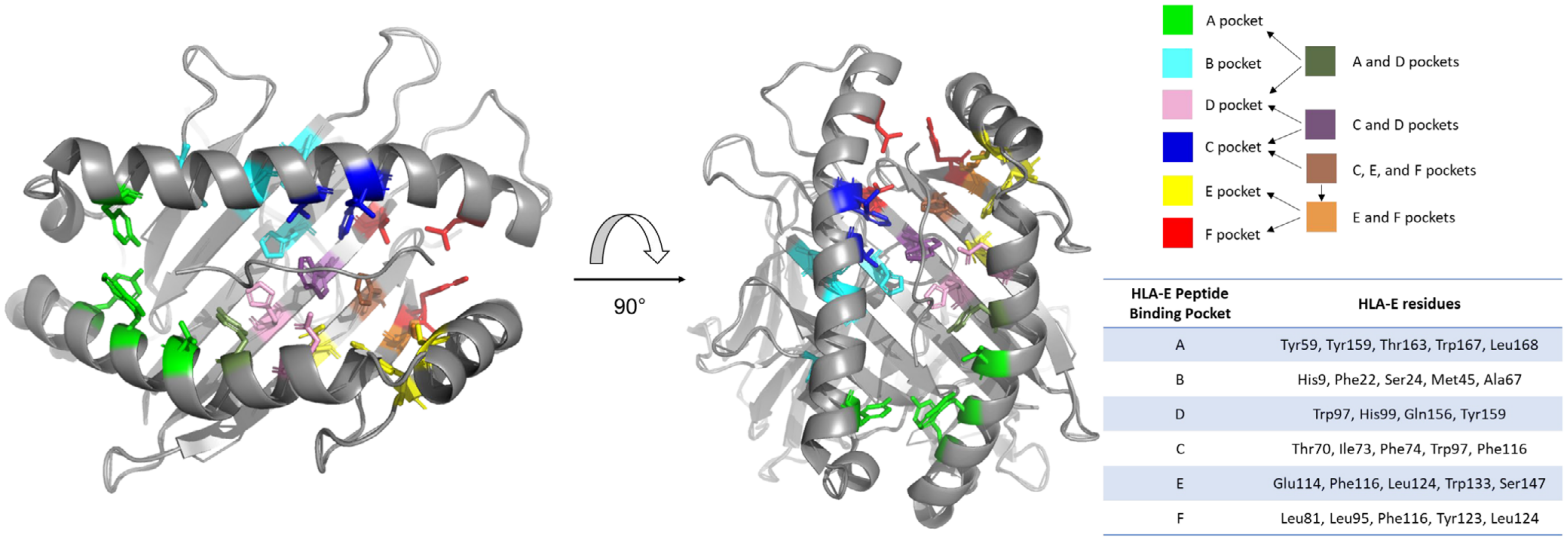
The peptide‐binding pockets of HLA‐E. Depiction of the peptide‐binding pockets of HLA‐E, color coded for the different pockets: green (A pocket), cyan (B pocket), pink (D pocket), blue (C pocket), yellow (E pocket), and red (F pocket). For residues which are part of more than one peptide‐binding pocket, these are colored based on the combination of the colors for the associated pockets. A key is provided along with a key listing the residues associated with each peptide‐binding pocket.

HLA‐E and classical MHC class I complexes share many similarities, particularly in terms of their overall structure. The length, width, and depth of the HLA‐E peptide‐binding groove (PBG) is of standard proportions, and like classical MHC class I proteins, a number of small cavities along the length of the groove create pockets accommodate the side chains of the bound peptide. HLA‐E contains five well‐delineated peptide‐binding pockets (B, C, D, E, and F). This marks a substantial difference between HLA‐E and the majority of classical MHC class I types [46]. The primary B and F pockets accommodate the peptide's side chains at positions 2 and 9, respectively, whereas the D, C, and E pockets form the secondary cavities that for the leader sequence peptide VL9 (VMAPRTLLL or equivalent) are occupied by side peptide chains at the respective positions 3, 6, and 7 (Figure 1). Similar to classical MHC class I, the peptide N and C termini are tethered to conserved resides at either end of the groove that maintain both main and side chain interactions to stabilize peptide binding to HLA‐E [46].

Aside from the employment of additional peptide‐binding pockets, other distinguishing features are noteworthy. The first relate to hydrogen bonding network that stabilizes the bound peptide to HLA‐E. The second reflects differences in the elevation of the peptide—noted specifically for leader sequence peptides—toward the C‐terminus end of the HLA‐E PBG.

For the original HLA‐E‐VL9 x‐ray crystal structure (HLA‐E*01:01‐VMAPRTVLL [PDB: 1MHE]), seven hydrogen bonds between the peptide backbone at positions 1, 2, 5, 7, and 9 and the HLA‐E α‐helical residues were described [46]. Those mediated between the N and C termini of the peptide and the A and F pocket regions are conserved between HLA‐E and classical MHC class I. A key interaction involving a conserved hydrogen bond between the hydroxyl groups of the α1 helix Tyr84 residue and the α2 helix Thr143 and the peptide's terminal carboxylate group is noted for all MHC class I bound peptides crystallized to date; despite the substitution of Thr143 for Ser in HLA‐E, this hydrogen‐bonding network is maintained. However, adjustments to more centrally positioned hydrogen‐bonding networks are evident. For nearly all MHC class I, an additional, highly conserved interaction between Trp147 and the carbonyl oxygen of the peptide's penultimate residue further stabilizes peptide binding. Together with an interaction between Lys146, these conserved interactions stabilize the peptide in the MHC class I PBG. The replacement of the conserved Trp at position 147 for Ser in HLA‐E creates an enlarged and distinct E pocket cavity, disrupting the normal stabilizing bond between position 147 and the peptide main chain at position 8. However, an alternative set of peptide‐stabilizing hydrogen‐bonding networks exists for HLA‐E, including two hydrogen bonds formed between Asn77 on the α1 helix with the VL9 peptide main chain at positions 7 (carbonyl oxygen) and 9 (amide nitrogen). For HLA‐E‐bound VL9, a further hydrogen bond is formed between the VL9 peptide main chain position 5 Arg and the side chain of Gln156 via its amide nitrogen. Additionally, a salt bridge between the same peptide position 5 Arg side chain and the Glu152 has also been reported for some HLA‐E–VL9 structures. As discussed later, this interaction between the peptide position 5 main chain and HLA‐E Gln156 is absent in almost all of the non‐VL9 peptide–HLA‐E structures described to date.

Two HLA‐E structures in complex with non‐VL9 peptides were reported by our group in 2018 [45]. The first included a strong HLA‐E binding peptide from *Mycobacterium tuberculosis* (Mtb) known as Mtb44 (RLPAKAPLL) [27]. The conformation of Mtb44 in the HLA‐E PBG displayed strong similarity to VL9: Mtb44 adopted the classical VL9 peptide kink and shared eight of the nine hydrogen bonds between the peptide and the HLA‐E heavy chain. The B pocket forming residues were minimally altered by the Mtb44 position 2 Leu, and the shallow C and D pockets were occupied by the peptide position 3 Pro ring and the position 6 Ala side chain, respectively.

The second structure of the much weaker binding HIV‐1 gag‐derived “RL9H” peptide (RMYSPTSIL) exhibited a structural trajectory highly distinct to Mtb44‐ and VL9‐bound HLA‐E complexes. A major shift of the peptide “kink” was facilitated by the lack of C and E pocket occupancy by the RL9H peptide positions 6 and 7 residues, respectively. Additionally, the smaller D pocket failed to accommodate the large side chain of the peptide position 3 Tyr; instead, the Tyr side chain formed a hydrogen bond to the HLA‐E α2 Glu 152 residue. Although six of the nine bonds formed between RL9H and HLA‐E were conserved, four novel bonds were formed, with one between Glu152 and the peptide backbone at position 7, contrasting HLA‐E‐VL9 complexes where the position 7 main chain interaction is instead mediated by Asn 77 on the HLA‐E α1 helix. The reduced pocket occupancy that characterizes RL9H binding to HLA‐E may explain the dramatically reduced half‐life of binding [20], and as discussed later, could have a substantial impact on the configuration of the α2 helix near the C terminus of the PBG.

We reported two additional structures of HLA‐E in complex with Mtb derived peptides in 2022. The first is the Mtb14 peptide (RMAATAQVL), that like VL9 and Mtb44, adopted a similar, centrally positioned, solvent‐exposed kink in the HLA‐E PBG. However, by comparison, Mtb14 displayed greater mobility, especially toward peptide positions 4 and 5. This was contributed by a number of factors. First, Mtb14, lacks the typical peptide di‐motif “Pro–Ala” or “Ala–Pro” at peptide positions 3 and 4, observed, respectively, for VL9 and Mtb44 peptides. The Ala at position 3 of VL9 confers the preferred anchor residue that is accommodated by the shallow D pocket, and though a Pro residue at this position, as in the case of Mtb44, sits minimally within the pocket, it provides rigidity to the peptide by restricting movement. Second, Mtb14 lacks the preferred anchor residue at position 7, and though the peptide position 7 Gln side chain is accommodated in the large, hydrophobic E pocket, it is likely that an entropic penalty is associated with the water‐mediated hydrogen bond formed in this predominantly hydrophobic cavity. Collectively, the lack of Pro residue coupled to nonoptimal occupancy of the E pocket by the position 7 side chain and the lack of the typical peptide position 5 main chain HLA‐E Gln156 interaction culminated in the weaker binding of Mtb14 to HLA‐E.

The Mtb‐derived peptide, MtbIL9 (IMYNYPAML), originally identified when eluted from Mtb‐infected macrophages [16] also offered an intriguing picture of dual binding peptide modalities for the same peptides—made apparent by virtue of the asymmetric unit (ASU) providing four molecules, with two molecules in noncrystallographic symmetry providing two different configurations of the peptide. The alternative peptide configuration was focused to the region of the E pocket. In the first orientation, observed for two of the four molecules, MtbIL9 strongly resembled VL9, in that the peptide position 7 Ala sat relatively deep in the E pocket. In the second orientation, the Ala points upward, out of the E pocket, and projected toward the α2 helix. By comparison, the Cα backbone of Ala sits over 2 Å higher than the equivalent Ca atom of the VL9 position 7 anchor residues. Why these dual conformations were formed is unknown, but it seems likely that the shallow fit of the position 7 Ala side chain or the adjacent Pro residue—or the combination of both—impacted binding and facilitated the conformations captured here.

Collectively, these structural studies highlight the range of mechanisms employed by HLA‐E to bind peptides that lack the full complement of primary and secondary peptide residues, ranging from the stabilizing effects of central Pro residues to the “out‐of‐pocket” binding mode observed for two peptides containing a position 3 Tyr. It will be of interest to elucidate if other peptides utilize further modes of interactions to facilitate HLA‐E binding.

## 5 From Peptide‐Free HLA‐E Refolding to PBG Stability

HLA‐E shows a remarkable ability to both form a complex with β2m in the absence of added peptide, and to remain in a peptide receptive state in vitro. These features, however, are not wholly unique to HLA‐E, and “peptide devoid”/peptide receptive forms of certain human and murine classical MHC I types have been previously described (reviewed in Ref. [47]). However, HLA‐E‐β2m dimers that are “peptide free” appear remarkably stable compared to the heretofore studied human MHC class I‐β2m “peptide‐free” complexes. In cellular‐based studies, HLA‐E has also been reported to exist at the cell surface as a β2m‐bound, peptide‐devoid form [48]. Most of what is currently known about "peptide‐free" complexes come from the study of human and murine classical MHC class I types. For human HLA‐B*07:02 refolded without peptide, studies in the late 1990s identified an exposed protease cleavage site in the region of the α1/ α2 helices that delineated the PBG, with the authors concluding these helices are only partially folded without peptide compared to the peptide‐distal α3 domain [49]. More recent studies of “peptide‐free” HLA‐Cw7 forms, using nuclear magnetic resonance (NMR) and hydrogen–deuterium exchange/mass spectrometry (HDX/MS) also suggest that the α1 and α2 helices are poorly structured compared to the α3 domain [50]. Studies of murine H‐2 Kb using Fabs that specifically recognizes peptide‐free (open) forms suggest "peptide‐free"/peptide‐receptive partially folded forms exist at the cell surface [51].

However, “peptide‐free” HLA‐E‐β2m dimers are easy to produce by refolding in vitro, and at least when compared to human MHC class I subtypes, appear by comparison to be unusually stable. Yet this increased stability does not overtly equate to higher affinity for β2m, as Tm comparisons for peptide‐free HLA‐E‐β2m versus MHC I‐β2m are comparable. In fact, despite the remarkable yield of Mamu‐E–Mamuβ2m complexes refolded without peptide—by comparison up to 10‐fold over that of refolded HLA‐E‐β2m—the thermal stability of the co‐complex compared to HLA‐E is lower. The stability of the dimer is instead most likely linked to intrinsic qualities specific to the HLA‐E and Mamu‐E heavy chain sequences. What conveys these qualities?

Compared to other MHC class I types, the HLA‐E PBG is dominated by an aromatic, hydrophobic signature. A number of key amino acids most notably those at positions 74 (Phe), 97 (Trp), 99 (His), 116 (Phe), 143 (Ser) and 147 (Ser) are unique to HLA‐E [46]. These mostly include an enrichment of aromatic substitutions that help shape the peptide‐binding pockets. Phe74, alongside Trp97 and Phe116, defines the lower C pocket region that accommodates the peptide's position 6 side chain of VL9 in a shallow and predominantly hydrophobic recess. In MHC class I heavy chains, position 74 is generally represented by an Asp, Tyr, or His residue. Also unique to HLA‐E is Trp97, which helps form the D pocket that accommodates the peptide position 3 side chain. This residue alongside Tyr159 and His99, the latter also unique to HLA‐E, contributes to the smaller pocket size and its hydrophobic character. Position 97 is generally polar in classical MHC class I proteins. Interestingly, the murine H2‐Kb and H2‐Kd heavy chains that readily form dimers with β2m in the absence of peptide also contain a Phe residue at position 74 and either Tyr or Phe (the latter shared with HLA‐E) at position 116. They, however, lack an aromatic residue at position 97. Indeed, the unique combination of His9–His99–Trp97 in the HLA‐E PBG was previously recognized and discussed, particularly in relation to the possibility that Trp97 could increase the pKa of nearby His9 to facilitate its protonated stage and thus facilitating a hydrogen bond between His9 and His99 [46]. If this and the other amino acid combinations contribute to the stability of peptide‐free HLA‐E is currently unknown, however, the hydrogen‐bonding networks alongside the large aromatic side chains of these amino acids could collectively provide a stabilizing structural framework permeating the length of peptide‐free HLA‐E PBG.

Other regions of interest—namely regions of flexibility—based on the findings from the study of classical MHC I are noteworthy, especially if these regions differ with respect to their stability in HLA‐E. The C terminal of the PBG in the vicinity of the α2‐1 helix, for example, has been shown by molecular dynamic simulations (MDS) for a number of MHC class I types to be highly flexible in the absence of peptide [52]. Interestingly, HLA‐E uniquely differs from classical MHC class I in this region, which is in part related to the substitution of Ser residues at positions 143 and 147, in contrast to the conserved Thr and Trp residues, respectively, present in classical MHC I molecules. The lack of Trp147 in HLA‐E removes a hydrogen bond to the main chain of peptide's penultimate amino acid and also generates a cavity to form the large E pocket that is unique to HLA‐E. Our previous x‐ray structural work displayed α2‐1 helix movement for peptides with position 7 side chains that failed to optimally occupy the E pocket [41, 45]. Similarly, more extreme deformations of the HLA‐E α2‐1 helix were observed when peptides were stabilized through a disulfide bond involving the introduction of a Cys residue at position 84 of the α1 helix, in contrast to the modest movements observed for equivalent changes to classical MHC class I, which supports the likelihood of greater mobility in this region for HLA‐E [20]. It has been suggested that for MHC class I, fixation of this region might be required to stabilize the protein and this idea is supported by studies using truncated peptides that specifically stabilize the F pocket [53]. However, HLA‐E appears to refold “empty” and this may, in part, be due to the unusual mobility of the α2‐1 helical region, most likely contributed to by its unique sequence composition.

A second region of mobility, identified during MDS‐based analyses of human HLA‐C, maps centrally to residues 66–76 on the α1 helix [54]. Interestingly, HLA‐E uniquely differs to all classical MHC class I types in this region. Whether this region provides an element of structure and ultimately peptide‐free stability to the HLA‐E PBG warrants investigation. A study of murine H2‐Ld has also mapped areas of flexibility to the N terminus of the α1 helix, namely the 3–10 helix [55], and in particular the conserved Trp side chain that contributes to the formation of the A and B pockets, adopting a different state in peptide‐free forms [56]. A final region includes the peptide‐distal α3 region of MHC proteins. It has been shown for MHC class I that this region undergoes rigid body movement upon peptide binding. Dramatic, peptide‐dependent rigid body shifts in the α3 region were previously observed for the different HLA‐E complexes solved in our laboratory, signifying the different impact of the bound peptides on the α3 domain configurations [41]. While these movements could in part reflect crystal packing artifacts because of greater mobility in solution, the reconfigurations were exaggerated compared to the movement described for classical MHC I α3 regions. And while recent evidence using α3 domain swap mutants suggests that this region is not required to produce "peptide‐free", peptide‐receptive HLA‐E‐β2M dimers, the α3 domain of HLA‐E could signify a region of interest in relation to understanding peptide editors [57] and the possible role played by tapasin, if any, for the binding of non‐VL9 peptides.

The biological significance of stable β2m‐HLA‐E dimers that are peptide receptive is currently unknown. Perhaps similarly stable dimers exist in vivo where upon recycling to endocytic vesicles they acquire peptide cargo. Or perhaps the intrinsic qualities of the HLA‐E heavy chain also allows it to remain receptive to β2m rebinding, thereby creating a population of dimeric material that can be loaded with peptide cargo in an endosomal–lysosomal recycling pathway. Further studies are warranted to connect the significance of in vitro observations to in vivo functions.

## 6 The Unusual Structural Dynamics of HLA‐E Peptide Loaded Forms

We recently combined biophysical, biochemical and structural studies to interrogate the binding of pathogen‐derived peptides, previously reported to be immunogenic, to HLA‐E. Despite the low thermal stability and fast peptide off‐rate of all but one of these peptide epitopes [20], we obtained crystal structures for five of the six peptide‐HLA‐E complexes under investigation [41]. We employed size exclusion chromatography coupled small angle x‐ray scattering (SEC‐SAXS), a low resolution, in‐solution‐based x‐ray scattering technique where proteins are exposed to x‐ray during size‐based elution. The method provides insight into a number of factors including overall protein shape, dimensions, conformations, order/disorder, and structural transitions. Using SEC‐SAXS, we observed that in contrast to VL9, HLA‐E folded with weaker binding pathogen‐derived epitopes formed large, conformationally heterogeneous HLA‐E protein ensembles in solution. This reflected prior blue native gel evaluation of HLA‐E‐peptide complexes where compact bands were observed for stronger binding peptides in contrast to diffuse bands for weak binders [45]. The biophysical SEC‐SAXS measures obtained, Dmax (maximum dimensions) and volume (V^3^), bore strong correlations with data generated independently using peptide‐binding assay techniques that collectively reinforced the weaker binding of these peptides to HLA‐E relative to the VL9 leader peptide [41]. Yet, when test peptides were maintained at high concentration throughout the entirety of SEC‐SAXS runs, compact and homogeneous HLA‐E protein forms that aligned closely to the x‐ray crystallographic coordinate dimensions of the same complexes were observed [41].

To elucidate if the x‐ray structural data could help shed light on our SEC‐SAXS‐based findings, we compared all previously published HLA‐E‐VL9 structures to our non‐VL9 HLA‐E–peptide complexes. These comparisons uncovered two key structural aspects that separated VL9 from the other HLA‐E–peptide complexes. The first mapped to the E pocket of HLA‐E where nonoptimal occupancy by the peptide side of all non‐VL9 peptides tracked with conformational heterogeneity identified by SEC‐SAXS. This large pocket is well formed and discrete, and its hydrophobic character and depth are contributed by Phe116, Tyr133, and Ser 147. Ser147 is unique to HLA‐E and contrasts classical MHC I proteins where a universally conserved Trp147 largely restricts access to this pocket. The Leu or Val side chain residues present in VL9 peptides are optimally accommodated by this pocket. This, however, contrasted the weaker binding peptides where the pocket was either minimally occupied, as in the case of the position 7 Ala side chain of the MtbIL9 peptide (IMYNYPAML), suboptimally occupied by a destabilizing polar residue side chain as for the Mtb14 peptide position 7 Gln (RMAATAQVL), or not at all as displayed by the RL9H (RMYSPTSIL) peptide when the position 7 Ser side chain projected high into the solvent. The second finding related to a novel structural configuration generated exclusively for non‐VL9 binding that centered on the α2 helix of HLA‐E. Central to this was the α2‐residue Gln156 that in the case of HLA‐E‐VL9 complexes formed a hydrogen bond with the peptide Arg main chain at position 5. For most HLA‐E‐VL9 crystallographic complexes a salt bridge between the peptide position 5 Arg and Glu152 on the HLA‐E α2 helix has also been observed. Yet in all non‐VL9 bound complexes, the hydrogen bond between the peptide main chain at position 5 and HLA‐E Glu156 side chain was absent. The only slight exception was the strong binding Mtb44 where the central position 5 main chain hydrogen‐bonding interaction was observed in two of the four molecules in the ASU. For most of the weaker binding peptides, the Glu152 side chain also adopted an alternative side‐chain orientation compared to the VL9 peptide structure, and for the MtbIL9 and RL9HIV peptides, generated a structural configuration that facilitated “out‐of‐pocket” binding to the position 3 Tyr residues of those peptides to Glu152.

**FIGURE 2:**
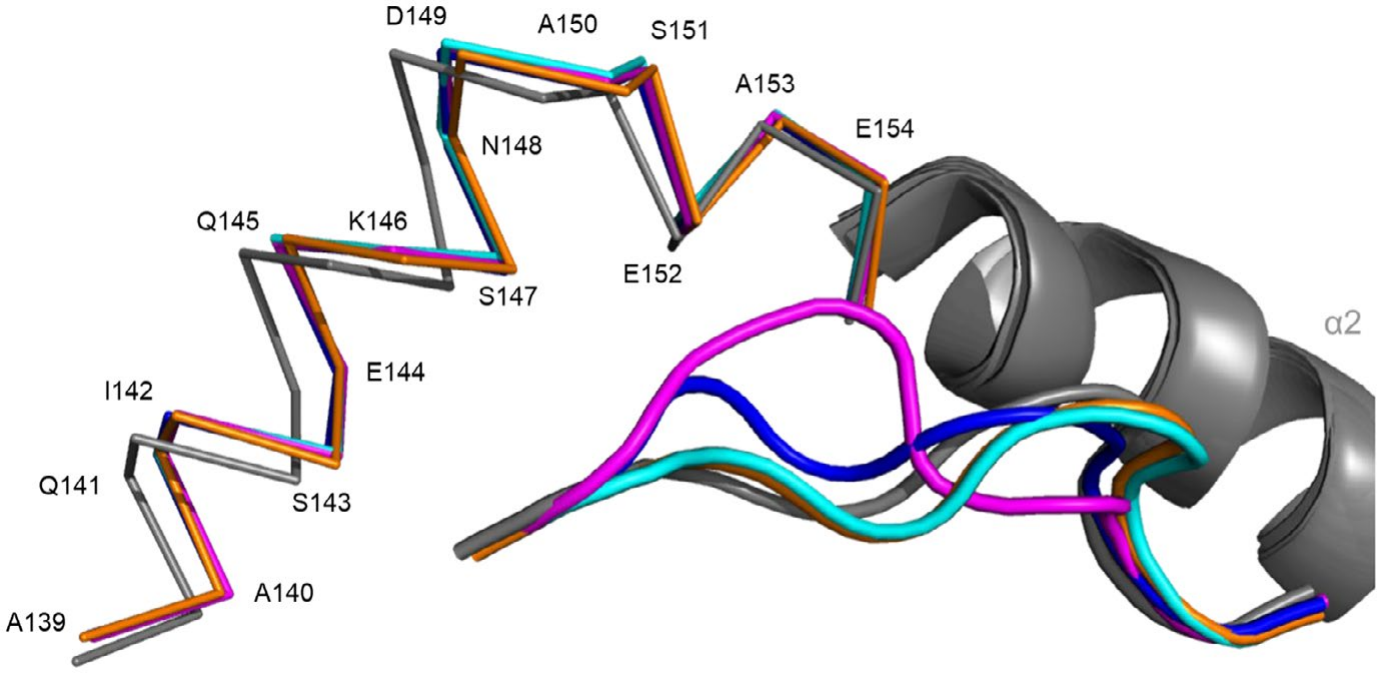
A comparison of the α2‐1 region of HLA‐E with VL9 compared to current non‐VL9 peptide structures. An alignment of HLA‐E VL9 structure (gray, PDB ID 1KPR) compared to non‐VL9 structures, Mtb44 (orange, PDB ID 6GH1), RL9H (magenta, PDB ID 6GL1), MtbIL9 (blue, 7P4B), and Mtb14 (cyan, PDB ID 7P49), shows a shift in the α2‐1 region of HLA‐E starting at position A139. The section of the α2 which is conserved between structures is depicted in gray (β sheet removed for clarity).

Reorientation of Glu152 and neighboring amino acids, coupled with the lack of the highly conserved and stabilizing peptide position 5 main chain interaction with the Gln156, generated regional movement and a local α2 structural reconfiguration (Figure 2). Collectively, these adjustments formed the basis for an alternative immune recognition surface that distinguished HLA‐E‐VL9 and non‐VL9 bound peptides. This surface reconfiguration which is shared to some extent for different pathogen‐derived peptides might help explain the unexpected TCR‐mediated cross‐reactivity to sequence‐disparate peptides recently described for Mamu‐E [58]. Although the x‐ray crystal structures provide clues as to why these peptides bind weakly to HLA‐E, they fail to shed light on the composition of the heterogeneous molecular ensembles identified by SEC‐SAXS analysis. The x‐ray structures represent a specific subpopulation of peptide–HLA‐E complexes, the most likely form being the lowest energy state. In contrast, the SAXS data exemplifies the average conformations in solution, including partially unfolded or flexible intermediate transition states that exist alongside the lowest energy form represented in static crystals. So, what specifically, are these heterogeneous intermediates? As the SEC peak specifically captures the HLA‐E‐β2m‐peptide monomer, the ensembles likely represent β2m‐bound HLA‐E forms in different conformational states caused by weak binding peptides. So what might these various states represent? Do they comprise a combination of peptide folded and peptide‐free, partially unfolded HLA‐E‐β2m dimeric forms? And if containing unfolded regions, is this confined to a specific region, perhaps the α1 and α2 helices? And within peptide‐bound states, to what degree is the peptide bound suboptimally in the groove partially open, hydrated form with partially bound peptide, or as a closed dehydrated form following optimal peptide binding akin to the transition forms hypothesized previously for HLA‐B*35:01 based on data generated from NMR studies [59].

## 7 HLA‐E, Tapasin, and Peptide‐Binding Repertoires

It is widely accepted that MHC–peptide complexes are dynamic structures, a feature most likely required to allow rapid sampling of peptide repertoires. However, the extent to which different MHC achieve rapid peptide sampling varies, with many subtypes requiring help from the peptide editor, tapasin. Tapasin functions as a both chaperone and peptide editor for a number of MHC class I allotypes [60]. For tapasin‐dependent allotypes, it has been proposed that they display poor ability to self‐select high‐affinity peptide cargos, hence this peptide editor enhances peptide sampling by increasing the PBG closure rate when in complex with optimally bound peptides. In contrast, MHC types that are less dependent on tapasin display dynamic properties allowing them to self‐optimize peptide repertoires [61, 62, 63]. Interestingly, HLA‐E has been reported to show reliance on tapasin to present VL9 peptides, and HLA‐E surface expression is higher in tapasin expressing cells [64]. Yet the extent to which tapasin impacts the binding of non‐VL9 peptides is currently unknown, though studies of Qa‐1b suggest that its peptide repertoire diversifies when components of the peptide loading complex including tapasin and TAP are downregulated [65, 66]. Whether the same holds true for HLA‐E and Mamu‐E remains unknown.

Most of what is known about tapasin comes from the study of MHC class I allotypes. One obvious feature is that they tend to have a stable F pocket conformation, contributed by the presence of an aromatic amino acid residue at position 116. An elegant study by Abualrous et al. [67] compared the conformational stabilities of the related subtypes HLA‐B*27:05 and HLA‐B*27:09 to understand their different dependencies on tapasin. Using MDS, the authors mapped the largest width variation to the PBG, and specifically the F pocket region of peptide‐free HLA‐B*27:05, the most tapasin dependent of the two HLA‐B*27 subtypes. They further demonstrated that without peptide, the acidic residues Asp116, Asp77, and Asp74 in the HLA‐B*27:05 PBG create a negative electrostatic potential, causing repulsion and instability in the vicinity of the F pocket. In contrast they reported that the single F pocket mutation at position 116 distinguishing HLA‐B*27:09 (His116) from HLA‐B*27:05 (Asp116) would help neutralize the negative electrostatic potential and provide greater conformational stability (and hence a lower dependency on tapasin) in this region. It was postulated that the requirement for tapasin might stabilize the peptide empty form in the absence of high‐affinity peptide. Similar to other MHC class I types that are least reliant on tapasin, HLA‐E has a bulkier Phe residue at 116. A second study evaluated the plasticity of tapasin‐independent HLA‐B*44:05 and mapped important interactions in the α2, β2m, and α3 regions that collectively define the footprint of the interaction of tapasin with MHC. They observed that reliance on tapasin was significantly reduced by mutating the E pocket Trp147 to an Ala amino acid. Interestingly, the E pocket of HLA‐E contains an exclusive Ser at position 147. It is also noteworthy that HLA‐E carries a number of amino acids that are distinct to those that map onto MHC class I in the tapasin binding interface. So, the extent to which HLA‐E relies on tapasin is unclear at present. One possibility is that the unusual stability of peptide‐free HLA‐E‐β2m dimers might create an opportunity for the acquisition of low‐affinity‐binding peptide cargos**—**perhaps this is elevated in tapasin‐devoid compartments, for example, when in endolysosomes as suggested for both HLA‐E and Mamu‐E [4, 36, 58] or when tapasin is downregulated in the ER as shown for Qa‐1b [65] to promote the loading of low‐affinity peptide cargo. And does this allow HLA‐E to exist in a state of peptide‐sampling flux, generating non‐native HLA‐E conformations when with weak binding peptides, and if they exist, to what extent, if any, are these forms recognized by T cells? Further studies to address these questions are warranted.

## 8 T‐Cell Restricting Peptides That Register Biochemically as Non‐MHC‐E Binders

A number of peptide epitopes [25, 27] that are reportedly recognized by T cells via MHC‐E restriction show unreproducible or undetectable binding using our current peptide‐binding methods [40]. These peptides lack all preferred anchor‐binding residues and sequence‐binding motifs predicted using public servers or in‐house binding algorithms [25, 27]. This highlights a puzzling disparity between the published T‐cell‐based data and our binding studies. So, what underlies the ability of these peptide to bind MHC‐E? One possibility is that the binding of these peptides is somehow facilitated in vivo, but what exactly could facilitate their binding? A precedent for peptide binding mediated by small molecules exists for certain human MHC class I allotypes. For example, small drug compounds that occupy the PBG are known to drastically alter the bound‐peptide cargos. The nucleoside reverse transcriptase inhibitor Abacavir when given to people living with HIV (PLWH) as an antiretroviral causes life‐threatening drug sensitivities in individuals who carry HLA‐B*57:01 [68]. This condition is mediated by drug‐induced changes to the HLA‐B*57:01‐bound peptide repertoire resulting in novel T‐cell‐mediated hypersensitivity directed against the drug‐altered peptide repertoire. Abacavir forms a noncovalent bond in the HLA‐B*57:01 PBG, with the cyclopentyl and purinyl drug moieties sitting in the vicinity of the D and E pockets, respectively, and the cyclopropyl ring near the F pocket [69]. A number of residues, particularly those at positions 74, 97, 99, 116, and 124 are key to this interaction, and explain why the specificity is unique to HLA‐B*57:01 and not observed for the related HLA‐B*57:03 subtype that differs by only two amino acids (at positions 114 and 116) in this region. A similar drug sensitivity has been described for HLA‐B*58:01 and the xanthine oxidase inhibitor drug, allopurinol, prescribed for gout [70]. Although the molecular mechanism underlying this pathology is not fully understood, there is recent evidence suggesting that this drug also alters the peptide repertoire presented by HLA‐B*58:01 [71]. The final example relates to carbamazepine and HLA‐B*15:02 [72]. This antiepileptic drug increases the hydrophobicity of peptide bound to HLA‐B*15:02; it reportedly shifts the B and F pocket peptides anchor preferences and allows the accommodation of small peptide residues at peptide positions 4–6 [72]. Although the site of carbamazepine binding is unknown, in silico predictions suggest that this maps to position 156 on the α2 helix under the central portion of the bound peptide. Nondrug‐related examples of facilitate binding have also been described for MHC proteins. Diamino acids, for example, stabilize the PBG E and F pocket regions for certain classical MHC I and allow additional peptide fragment cobinding [73]. There are also examples, for nonclassical MR1 proteins, of bacterial derived riboflavin metabolites that either by themselves [1] or when combined with small by‐products of glycolysis [74] serve as direct ligands for mucosal‐associated invariant T (MAIT) cells. In the case of MHC‐E, it is likely that that if present, small metabolites are not directly recognized by T cells, but instead could allow peptide binding in vivo by forming a conduit between apparent “nonbinding” peptides and MHC‐E.

## 9 HLA‐E–Peptide Receptors Structures

**FIGURE 3:**
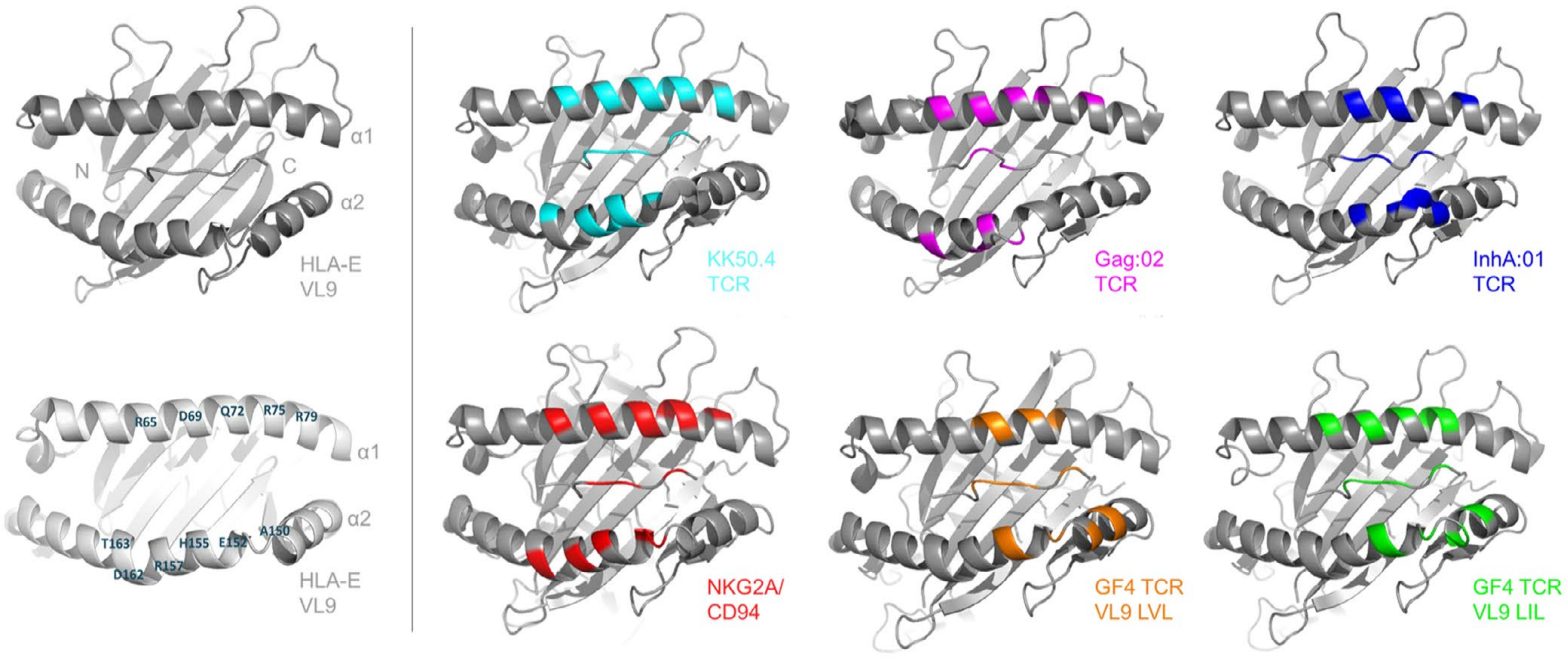
The binding footprint of NKG2A/CD94 and HLA‐E‐specific TCRs. Structures of the HLA‐E surfaces with the receptor‐binding footprints are shown. For reference, the structure for HLA‐E VL9 (PDB ID 3BZE) and labeled alpha helix residues are noted (left, upper and lower, respectively). To left are the binding footprints (at distances within 3.5 Å) for NKG2A/CD94 (red, PDB ID 3CDG), GF4 TCR with VL9 peptide VMAPRTLVL (orange, PDB ID 5W1W), GF4 TCR with VL9 peptide VMAPRTLIL (green, PDB ID 5W1V), KK50.4 TCR (cyan, PDB ID 2ESV), Gag:02 TCR (magenta, PDB ID 7NDQ), and the InhA:01 TCR (blue, PDB ID 6ZKW).

The structures of HLA‐E–VL9 peptides in complex with NKG2A and T‐cell receptors are elegantly summarized elsewhere [75], the salient features are presented again here (see Figure 3 for general footprint). Two separate crystallographic structures of the inhibitory CD94‐NKG2A heterodimer in complex with HLA‐E bound to the HLA‐G leader peptide, VMAPRTLFL, have been reported [76, 77]. In relation to the CD94/NKG2A‐HLA‐E VL9 (LFL) complex (PBD ID: 3CDG), the CD94‐NKG2A heterodimer docks in a diagonal orientation with a slight bias to the C‐terminus end of the peptide. CD94 displays a broad binding footprint and dominates the interface, with two regions forming the main contacts to HLA‐E. Most notably, CD94 forms interactions along the α1 helix with residues encompassing positions 65–85, with specific points of contact made to Arg residues (at positions 65, 75, and 79), alongside a further contact involving Asp69. A separate contact to Glu152 on the α2 is also apparent. The NKG2A chain contributed less extensively, with the main interactions centered on HLA‐E α2 chain residues Ser151, His155, Ala 158, and Asp162. The CD94 chain also dominates the peptide recognition interface—peptide positions Arg 5, Thr 6, and Phe 8 formed the focus of the interface, and in particular, peptide positions 6 and 8, with three CD94‐contributed Asn residues at positions 156, 158, and 162 making contact to the peptide's position 8 Phe. Positions 156 and 158 are located on a loop that borders the peptide position 8 Phe residue, with mutagenesis analysis also suggesting that position 158 also forms an interaction with the peptide main chain at position 8 Phe [17]. The Gln112 of CD94 is also involved in binding to the peptide. By inserting into a gap between the 5 Arg and position 8 Phe of the VL9 peptide, this residue makes contact to the buried peptide backbone at the position 6 Thr residue. The script run for the purpose of this review (to capture bond lengths of 3.5 Å or under) also suggests that the CD94 Ser residue at position 110 interacts with the peptide position 5 Arg residue.

In contrast to CD94, the NKG2A chain forms a minor footprint with only a single hydrophobic contact to the peptide alongside a small number of contacts involving residues along the α2 helix region spanning amino acids 151–166 with Ser151 and His155 forming the main points of contact to positions Arg137 and Pro171/Ser172, respectively, of NKG2A.

CD94‐NKG2A binding to HLA‐E‐VL9 has been described as a rather fixed, “lock and key” mode of binding. This restricted mode of binding concurs findings from NK functional studies that reinforce the specificity of CD94‐NKG2A for VL9 or VL9‐like peptides in complex with HLA‐E [78]. VL9 positioning toward the α1 and the position 5 main chain stabilization, along with the optimal occupancy of the E pocket by the peptide position 7 side chain with the peptide‐specific requirements likely gives this tight specificity.

The activating NKG2C/CD94 receptor displays lower affinity than the closely related inhibitory NKG2A/CD94 receptor [17, 76]. Although there is no structural information available regarding NKG2C/CD94‐mediated recognition of HLA‐E, a mechanism resulting in the reduced binding affinity has been observed in a number of independent studies. Mutagenesis studies suggest that this maps to a region within a loop encompassing residues 167–170 that for NKG2A forms a key part of the heterodimer interface with CD94 [76]. Hence, it has been proposed that the affinity differences between these sequence‐related receptors is a likely consequence of adjustments to the CD94:NKG2C interface, leading to reduced binding to HLA‐E. In the absence of a CD94–NKG2C crystal structure, it is unclear if the functional discrepancies explained by subtle differences in specificities to different peptide repertoires is exaggerated by the different sensitivities of signaling modules for the activating and inhibitory signaling machinery [78].

## 10 The HLA‐E‐VL9‐Specific KK50.4 and GF4 TCRs

The crystal structures of two TCRs that interact with HLA‐E in complex with VL9 sequences have been described previously (see Figure 3 for summary of receptor footprints on HLA‐E). The first, known as the KK50.4 TCR (AV26‐1*01/BV14*01), recognizes a UL40‐derived peptide (VMAPRTLIL) variant that differed by a single amino acid to donor‐derived HLA leader sequence motifs (PDB:2ESV) [79]. Like many of TCRs that bind classical MHC I, the KK50.4 TCR docked diagonally to the long axis of the α1 ‐ α2 helices. The Ile at position 8 of the peptide formed the primary contact for the KK50.4 TCR, with all three CDR loops contributed by the TCRβ chain encasing the position 8 Ile side chain, though additional interactions (of 3.5 Å or under) are observed between both the TCR and the peptide at positions 4 (Pro), 5 (Arg), and 6 (Thr). The TCR CDR1α, CDR3α, and CDR3β chain segments all contribute to these peptide‐specific interactions. The TCR‐mediated HLA‐E‐directed contacts encompass residues 65–80 on the α1 helix and a network of residues from 154 to 165 on the α2 helix. The interface is dominated by polar interactions that permeate the entire interface.

The second receptor, GF4, is a BV9*01/AV35*02 TCR with specificity for the human CMV‐derived UL40‐derived VMAPRTLVL (PBD:5W1W), was identified in a donor lacking the sequence‐identical leader sequence motif [80]. Although the restriction of the TCR was identified against the VL9 epitopes ending “LVL,” this TCR also showed reactivity (albeit with 10‐fold lower affinity) against the VL9 peptides ending in “LIL.” Crystallographic structures of GF4 in complex with either the HLA‐E–VL9 (ending LVL) or the HLA‐E–VL9 (ending LIL) peptide (PBD:5W1V) demonstrated some notable differences to the KK50.4 TCR including an orthogonal mode of binding and a larger buried surface area interface compared to KK50.4. For the HLA‐E‐VL9 (LVL):GF4 structure, both the CDR3α and β loops centered on the peptide position 8 Val residue, with additional contacts between the CDR3α chain residues and the peptide position 4 (Pro), 5 (Arg), 6 (Thr), and between the CDR3β and the peptide position 5 Arg residue. The TCR‐mediated contacts to the HLA‐E α helices include TCR α‐chain‐mediated contacts to amino acids position on the α1 (position Asp 69) and α2 helical residues spanning regions 151–155. The TCR β chain‐mediated contacts were slightly more extensive and involve a stretch along the α1 helix encompassing residues 68–75 alongside further contacts on the α2 helix involving Gln 145 and Lys 146.

Although many of the HLA‐E‐restricted TCRs that we have worked on in our laboratory are potent against virally infected cells [34, 35], they appear to comprise low‐affinity receptors. This is suggested by poor HLA‐E tetramer staining, even when the TCR is expressed at high levels following transduction in T cell lines or on third‐party primary CD8+ T cells [34, 35]. The TCRs chosen for follow‐up have also proven challenging to produce as soluble receptors for affinity‐based studies. However, HLA‐E–TCR co‐crystal x‐ray structures have been solved for the Mtb44 and RL9H peptides—these TCR were selected from naïve TCR phage display libraries [20].

The first TCR—the inhA_53–61_‐specific TCR—recognizes the Mtb44 (RLPAKAPLL) peptide in complex with HLA‐E (PDB:6ZKW) [20]. A combination of charged and polar amino acids, most notably, Asp69, Gln72, and Arg79 on the α1 helix and Asp149, Glu152, and His155 on the HLA‐E α2 helix dominates this TCR:HLA‐E interface. Extensive contacts to consecutive peptide residues between positions 4 and 8 (namely Ala, Lys, Ala, Pro, and Leu) are mediated by the TCR CDR1α and CDR3α and the TCR CDR3β regions. A key peptide interaction dominates the interaction and centers on the peptide 5 Lys residue—this interaction was mediated by the formation of a recess contributed by three TCR α‐chain residues (Ala 29, Tyr37, and Gln109) and one TCR β‐chain residue (Arg111). This recess allowed what the authors termed a “knob‐in‐hole” mode of binding to the peptide position 5 Lys residue. Further contacts to the peptide at position 4 Ala and the position 8 Leu are mediated by the TCR CDR3β Arg111 residue, with another to position 8 Leu mediated by the TCR CDR1β Tyr 38 residue.

The second TCR—the Gag:02 TCR—recognizes the RL9 HIV C‐clade variant peptide RMYSP*V*SIL (so‐called Gag 6V_276–284_ peptide) in complex with HLA‐E (PBD:7NDQ) [20]. In contrast to the Mtb44 peptide, Gag 6V_276–284_ adopts a noncanonical orientation in the HLA‐E PBG where instead of occupying the C and E pockets, the positions 6 and 7 residues project into the solvent in a conformation identical to that described for the near‐sequence identical B‐clade peptide variant containing a position 6 Thr residue instead of Val [45]. This TCR engages a number of polar and charged α1 and α2 helical residues on the HLA‐E–Gag 6V_276–284_ peptide complex, most notably, the α1 residues Arg65, Asp69, Gln72, Arg75, and Arg79, and the α2 residues Glu152, His155, Arg157, and Asp162. Barber et al. [20] described a broad TCR specificity for the peptide, with a more even focus on multiple residues that centered on positions 1–4, 6, and 8, with extensive contacts mediated by a number of TCR CDR3α and CDR3β residues. These included TCR CDR3β residue‐mediated contacts to the peptide at the position 4 Ser and position 6 Val residues. As pointed out by the authors, these structures provide evidence that TCRs can bind HLA‐E–peptide complexes using disparate binding modes akin to examples described for classical MHC class I. However, as mentioned previously, the evolved TCRs used in this study originated from a phage library, hence it will be important to establish to what extent the binding modes utilized by these TCRs resemble the much weaker HLA‐E–peptide‐specific TCRs that appear to comprise the natural, post‐thymically selected repertoire that react against HLA‐E.

Remarkably, a pattern of shared contacts along the HLA‐E α1 and α2 helices were apparent, not only for the different TCRs with distinct peptide specificities, but also a number shared by CD94/NKG2A. The most prominent of these (and those with a bond distance of 3.5 Å or less) include α1 helix residue Asp69 and Gln72, and the α2 residue His155. It will be interesting to ascertain if the apparent low‐affinity receptors that recognized HIV and SARS‐CoV‐2‐derived peptides in complex with HLA‐E share similar chemical footprints as described here.

## 11 HLA‐E and Thymic Selection

There is a current lack of understanding how HLA‐E might shape T‐cell selection in the thymus. A few hypotheses are proposed here. One possibility is that low affinity TCRs are selected purely against HLA‐E‐VL9 or classical MHC I proteins, and subsequently cross‐react against pathogen‐presented peptides presented by HLA‐E in the periphery. In parallel, it is worth noting that there is a preexisting germline antibody repertoire with low‐affinity specificity for HLA‐E–VL9 [81]. A second hypothesis is that HLA‐E presents additional self‐derived peptides in the thymus that influence the restricted T‐cell repertoires. However, if lower affinity, the question remains of how these peptides would outcompete VL9, especially if abundance is low. If such peptides do indeed exist and bind, and as discussed earlier, in a manner that causes α2 helical reconfigurations distinct to VL9‐bound HLA‐E, the selected T cells could elicit broader cross‐recognition in the periphery. An insulin–peptide‐specific, Qa‐1b‐restricted TCR transgenic mouse model study provides an element of support to this hypothesis [82]. There may also be challenges when comparing the thymic education classically restricted to those that recognize HLA‐E in a more innate‐like manner, given that it remains unknown if all TCR–HLA‐E–peptide‐interacting domains fully map to nongermline‐encoded TCR αβ elements—such an example has previously been reported for a γδTCRs that binds non‐MHC ligands [83]. And, as shown for nonclassical MR1 complexes, the composite MHC–ligand surface is not always the primary recognition interface of the restricted TCR [84, 85]. The role of thymic selection in relation to HLA‐E–peptide‐driven TCR repertoire selection warrants further exploration.

## 12 Food for Thought: Murine H2‐Qa‐1b

Like HLA‐E, Qa‐1b has a broad tissue‐specific distribution [86], binds the leader Qa‐1‐determinant modifier (Qdm) peptide, AMAPRTLLL [11, 86], and participates in NK‐cell‐mediated immune surveillance using the CD94/NKG2A/C receptor system [87, 88]. T–cell‐mediated recognition of Qa1b has also been described, with examples including T cells expressing either αβ [89] or γδ [90] TCRs. A high‐resolution structure (1.9 Å) of Qa‐1b–Qdm was previously determined by Zeng et al. [91], and a few of the key feature, particularly features that compare and contrast HLA‐E, are noted here. Like HLA‐E, Qa‐1b employs five distinct PBG pockets to tether the Qdm peptide in the groove—these pockets precisely accommodate the same peptide side chains as described for HLA‐E (namely the position 2, 3, 6, 7, and 9 residues). The pockets are of similar size and chemical character to HLA‐E with the peptide position 2, position 7, and position 9 accommodating pockets B, E, and F, respectively, representing the deepest pockets, with shallower D and C pockets providing recesses, respectively, for the peptide side chains at peptide positions 3 and 6. As described for HLA‐E and classical MHC I types, the peptide is tethered in the end of the PBG by the N and C termini, via a conserved set of hydrogen bonds, between the Tyr7 and Tyr171 side chains and the peptide position 1 Ala, and the extensive hydrogen bond network anchoring of the peptide position 9 to the side chain of Tyr84, Ser143, and Lys146, among other water‐mediated bonds involving Asn77 and Thr80. A number of hydrogen bonds generated along the length of the peptide with α helical residues including the conserved hydrogen bond between the peptide Arg position 5 main chain and Gln156 and a salt bridge between the peptide position 5 Arg side chain and the Glu152 of Qa‐1b, as observed for HLA‐E‐VL9 structures.

**FIGURE 4:**
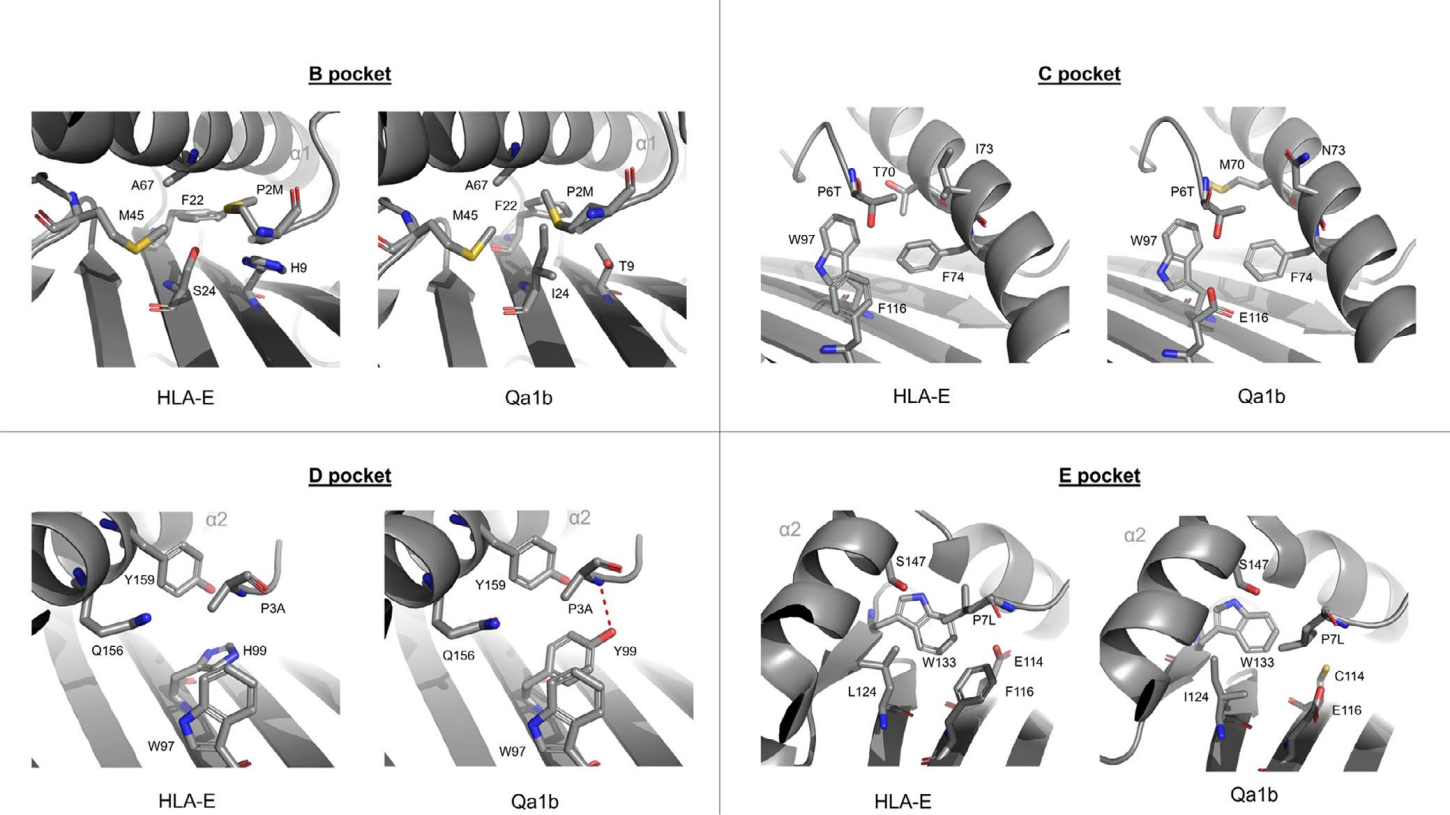
Comparison of the B, C, D, and E peptide‐binding pockets of HLA‐E and Qa1b. A comparison of the peptide‐binding pockets B (top left), C (top right), D (bottom left), and E (bottom right) for HLA‐E (PDB ID 3BZE) and Qa‐1b (PDB ID 3VJ6) complexes.

A couple of differences are noteworthy that center on amino acid substitutions specific to Qa‐1b (Figure 4). The first includes Lys66, and though forming a stabilizing bond to the peptide main chain at peptide position 2, this differs to HLA‐E where the position 66 Ser interaction to the peptide position 2 is mediated by a bridging water‐mediated contact involving the neighboring HLA‐E Glu63 residue. But the more striking difference maps to the Tyr99 residue that is also conserved in classical MHC types but is replaced in HLA‐E (and Mamu‐E) by a His residue. In the Qa‐1b–Qdm structure, Tyr99 forms a hydrogen bond to the main chain of the peptide's position 3 amide. It is likely that the lack of this bond in HLA‐E slightly weakens the strength of peptide binding to HLA‐E. Zeng et al. [91] argued that Qa‐1b‐specific interaction likely fixes the peptide positions 3 and 4 trajectories, in contrast to HLA‐E, where the smaller His99 replacement residue does not interact with position 3 of the VL9 peptide thereby allowing greater peptide mobility. Indeed, slight differences in the trajectories of the peptide at positions 3 and 4 for different VL9 peptides in complex with HLA‐E have been noted alongside a modest shift of the epsilon C atom of His99 [5, 46, 92]. We have noted substantial His99 side chain movement in the HLA‐E–RL9H (RMYSPTSIL) bound complex, most likely owing to the pocket void created by the “out‐of‐pocket” binding mode adopted by the peptide position 3 Tyr and the HLA‐E α2 Glu152 chain residue [45]. However, this contrasts the MtbIL9 peptide that despite adopting similar “out‐of‐pocket” peptide 3 Tyr—HLA‐E α2 Glu152 binding does not result in the reorientation of His99 residue side chain [45].

Other subtle differences exist that map to the larger B and C pockets. The B pocket, which supports the peptide position 2 side chain, has a similar hydrophobic character in HLA‐E and Qa‐1b. However, the Qa‐1b pocket is slightly shallower due to specific amino acid substitutions including Ile24, Lys66, Thr70, and Tyr99 residues. The authors suggested [91] that these changes likely restrict the movement and positioning of the Qdm position 2 Met in the binding pocket, evidenced in the structure by a slight shift of the side chain toward the α1 helix relative to HLA‐E. Another area of note is the E pocket, which is deeper in Qa‐1b due to the amino acid substitutions Glu116 and Ile124 that replace the HLA‐E‐specific Phe116 and Leu124 residues, respectively. As mentioned in that study, these changes likely allow both greater local flexibility and the accommodation of amino acids with larger side chains in this pocket. Finally, the F pocket of HLA‐E and Qa‐1b are remarkably conserved—the position 9 Leu orientation is highly similar in both due to the absolute conservation of sequence in this region. Despite the suggested differences, the few peptide elution studies for HLA‐E [16] and Qa‐1b [93] performed to date, both highlight the dominance of the respective leader sequence peptides, however, subtle differences in the Qa‐1b and HLA‐E peptides repertoires and especially in settings, should become evident with further studies in this area.

The lack of available x‐ray structures for Mamu‐E makes it impossible for comparisons to HLA‐E to be made. However, despite the existence of large number of Mamu‐E allotypes, they are all sequence identical in the PBG and are mostly identical to HLA‐E in all but a few α1/α2 changes. Of those that are noteworthy, the substitution of Ile73 in HLA‐E for Thr in all Mamu‐E allotypes might have significance—in human MHC I types, a Thr residue in this position often forms a hydrogen bond to the peptide main chain [94]. Whether this features and impacts the bound peptide repertoires by Mamu‐E will be important to explore.

## 13 How Does the Structural Biology of HLA‐E Relate to Its Cell Biology and Immunology

As described in the Introduction to this review, HLA‐E has unusual biological and immunological properties. It clearly evolved to regulate innate immunity by binding a highly conserved peptide VL9 from the signal sequence of classical MHC class I molecules and by presenting this to receptors on NK cells. Thus, it senses whether classical MHC I is present and its absence unleashes NK‐cell attack. HLA‐E‐VL9 by engaging NKG2A/CD94 monitors this. The weaker binding of HLA‐E–VL9 to the activating NKG2C–CD94 modulates inhibition, as does the short half‐life of HLA‐E–VL9 at the cell surface, giving a very sensitive balanced control. The obvious importance of this aspect of innate immunity in maintaining health has ensured the PBG of HLA‐E specifically binds VL9, almost to the exclusion of other peptides [16].

There has been increasing interest in the other peptides that can bind, albeit weakly, to HLA‐E and trigger more classical style T‐cell responses. These can be generated by priming T‐cell responses with HLA‐E binding peptides in vitro and the ability of these T cells to recognize pathogen‐infected cells indicates that small quantities of these peptides can access HLA‐E in living cells. Low frequencies of HLA‐E‐restricted T‐cell responses have been found in some acutely virus‐infected human donors if specifically sought [33, 35], though these are normally very subdominant to classical T‐cell responses. However, Hansen et al. [25] have shown that infection with a particular strain of rhesus CMV elicits these T‐cell responses in vivo in monkeys, excluding classical CD8+ T‐cell responses. *Mycobacteria* and *Salmonella* can also prime these T cells in humans [27, 29]. A possible common link between these disparate infections is the involvement of macrophages, which may handle pathogens in a unique way involving antigen processing in a series of endosomal compartments [36]. The relatively weak binding of VL9 and the stability of “peptide‐empty” HLA‐E may be important here in allowing this. HLA‐E‐bound VL9 delivered from the ER to the cell surface then subsequently endocytosed, may lose VL9 but retain β2m, and maintain sufficient stability and conformation to acquire novel peptides from an endolysosome compartment and then transport them back to the cell surface to prime T cells [4, 95]. Low‐affinity peptide binding to these relative stable empty forms could make this possible. However, this process is hard to reconcile with the fast off‐rates of peptides bound by HLA‐E; this remains a challenging puzzle to solve. Perhaps the abundance of antigen is key.

## 14 Translational Potential

The nonpolymorphism of HLA‐E combined with the immunological forces (NK‐cell inhibition) favoring upregulation when classical T‐cell responses have selected downregulation of classical HLA molecules makes HLA‐E and attractive target for immunotherapy. However, the low‐affinity binding of many peptides and conformational flexibility make HLA‐E difficult to work with [21]. Nevertheless, there is increasing evidence that MHC‐E‐restricted T cells are effective. While the combination of low‐affinity peptide binding with natural low‐affinity T‐cell receptors may not seem promising, the opposite could be the case. All TCRs are selected by the thymus for low‐affinity binding, in marked contrast to the complex mechanism that has evolved to mature antibodies by somatic hypermutation in germinal centers. T cells with laboratory‐selected high‐affinity receptors may work well in short‐term assays, but could also be very susceptible to exhaustion and activation induced T‐cell death and therefore be short lived in vivo. Also, artificial affinity enhancement is likely to produce some unwanted cross‐reactivities [96]. There may therefore be some advantage in following nature and to focus on effector T cells that can survive and function for many days or weeks in vivo using low‐affinity receptor:target interactions that may have evolved for just this purpose.

## Disclosure

All authors are coinventors of patents, owned by the Oxford University Innovation, that describe HLA‐E binding of peptides and the generation of HLA‐E–peptide‐specific T cells, T‐cell receptors, and antibodies.

## Conflicts of Interest

The authors declare no conflicts of interest.

## Data Availability

The data that support the findings of this study are available from the corresponding author upon reasonable request.
